# Haploid Germ Cells Generated in Organotypic Culture of Testicular Tissue From Prepubertal Boys

**DOI:** 10.3389/fphys.2018.01413

**Published:** 2018-10-09

**Authors:** Francesca de Michele, Jonathan Poels, Maxime Vermeulen, Jérôme Ambroise, Damien Gruson, Yves Guiot, Christine Wyns

**Affiliations:** ^1^Department of Gynecology-Andrology, Cliniques Universitaires Saint-Luc, Brussels, Belgium; ^2^Institut de Recherche Expérimentale et Clinique (IREC), Université catholique de Louvain, Brussels, Belgium; ^3^Institut de Recherche Expérimentale et Clinique (IREC), Centre de Technologies Moléculaires Appliquées (CTMA), Brussels, Belgium; ^4^Department of Clinical Biochemistry, Cliniques Universitaires Saint-Luc, Brussels, Belgium; ^5^Department of Anatomopathology, Cliniques Universitaires Saint-Luc, Brussels, Belgium

**Keywords:** prepubertal boys, testicular tissue cryopreservation, fertility preservation, *in vitro* maturation (IVM), haploid cells, human

## Abstract

While in mice various studies have described the completion of spermatogenesis *in vitro* using either organotypic culture of prepubertal testicular tissue or 3D culture of isolated cells, in humans it has not been possible to achieve germ cell differentiation from immature testicular tissue (ITT). In our study, we evaluated the ability of human ITT to differentiate via a long-term organotypic culture of frozen–thawed 1 mm^3^ testicular fragments from five prepubertal boys in two different culture media. Tissue and supernatants were analyzed at regular intervals up to day 139. Sertoli cell (SC) viability and maturation was evaluated using immunohistochemistry (IHC) for SOX9, GDNF, anti-Mullerian hormone (AMH) and androgen receptor (AR), and AMH concentration in supernatants. Spermatogonia (SG) and proliferating cells were identified by MAGE-A4 (for SG) and Ki67 (for proliferating cells) via immunohistochemistry (IHC). Apoptotic cells were studied by active caspase 3. To evaluate Leydig cell (LC) functionality testosterone was measured in the supernatants and steroidogenic acute regulatory protein (STAR) IHC was performed. Germ cell differentiation was evaluated on Hematoxylin-Eosin histological sections, via IHC for synaptonemal complex 3 (SYCP3) for spermatocytes, Protein boule-like (BOLL) for spermatocytes and round spermatids, angiotensin-converting enzyme (ACE), protamine 2 and transition protein 1 (for elongated spermatids) and via chromogenic *in situ* hybridization (CISH). We reported the generation of meiotic and postmeiotic cells after 16 days of culture, as shown by the histological analyses, the presence of differentiation markers and the increase of haploid germ cells. We showed SC viability and maturation by a decrease of AMH secretion in the supernatants (*p* ≤ 0.001) while the number of SOX9 positive cells did not show any variation. A decrease of spermatogonia (*p* ≤ 0.001) was observed. The number of apoptotic cells did not vary. LC functionality was shown by the increase in STAR expression (*p* ≤ 0.007) and a peak in testosterone secretion, followed by a reduction (*p* ≤ 0.001) with stabilization. According to our knowledge, this is the first report of generation of haploid cells in human ITT. Differentiating germ cells have to be further evaluated for their ability to complete differentiation, their fecundability and epigenetic characteristics.

## Introduction

It is well known that chemotherapeutic agents and radiotherapy have harmful effects on the gonads of prepubertal boys (Schrader et al., 2001; Wallace, 2011) which underlines the necessity to preserve fertility in these boys where generation of spermatozoa has not been started yet. One of the possible current options in terms of fertility preservation nowadays is the cryopreservation of immature testicular tissue (ITT) containing spermatogonial stem cells (SSCs) for future use when fertility restoration strategies, which are still under investigation, become available (Ginsberg et al., 2010; Wyns et al., 2011, 2015; Picton et al., 2015).

Different strategies have been studied, mainly in rodents, non-human primates and adult and prepubertal human testicular tissue to achieve completion of spermatogenesis from SSCs: autotransplantation of testicular tissue fragments or of one’s own selected germ cells (GC) and *in vitro* maturation (IVM) (for review see Jahnukainen et al., 2015; Onofre et al., 2016; de Michele et al., 2017b; Giudice et al., 2017; Vermeulen et al., 2017). So far, none of these approaches resulted in the generation of sperm using human testicular tissue. IVM has the objective to obtain spermatozoa that can be utilized for intracytoplasmic sperm injection, which makes this strategy the safest in terms of risk of reintroducing neoplastic cells back to the patient (Jahnukainen et al., 2001). Different techniques of IVM of GCs have been studied in both animals and humans, namely 2D and 3D culture of testicular cells, organotypic culture of testicular tissue, and the latest approach based on organoids (Galdon et al., 2016; Alves-Lopes and Stukenborg, 2017). In mice, since the first description of meiosis *in vitro* (Weiss et al., 1997), the entire process of spermatogenesis has been reported successfully (Dumont et al., 2015) with generation of offspring both from fresh and cryopreserved murine ITT (Sato et al., 2011; Yokonishi et al., 2014). In these reports, Knock-out Serum Replacement (KSR) 10% was used as culture medium, either with addition of FSH and testosterone (Sato et al., 2011; Yokonishi et al., 2014) or not (Dumont et al., 2015). By contrast, in humans, the completion of the spermatogenic process *in vitro* from ITT has not been achieved yet. It is however worth noting that spermatozoa-like cells were obtained after culture in chitosan bioreactors of seminiferous tubules retrieved from transsexual men which had impaired spermatogenesis leaving spermatogonia as residual germ cells in the tissue after hormonal therapy (Perrard et al., 2016). More recently, Sun et al., reported the differentiation of SSCs retrieved from azoospermic adult patients and cultured in a Matrigel system up to haploid cells able to fecundate mouse oocytes (Sun et al., 2018). All other attempts to *in vitro* differentiate human germ cells (GCs) exploited mature testicular tissue and only allowed the achievement of some steps of GC differentiation in organotypic (Steinberger et al., 1964; Tesarik et al., 2000b; Roulet et al., 2006), or 3D (Lee et al., 2006, 2007; Yang et al., 2014) culture. So far, organotypic culture seems to be the only technique -among the IVM strategies -able to preserve the testicular 3D architecture with intact paracrine interactions, and it is also the only approach that led to completion of spermatogenesis in mice (Sato et al., 2011, 2013; Yokonishi et al., 2013, 2014; Dumont et al., 2015).

This system has been applied to human prepubertal tissue in only two studies demonstrating the integrity of seminiferous tubules (STs) after short-term organotypic culture used to evaluate a cryopreservation technique (Curaba et al., 2011) and after long-term culture to achieve maturation of the prepubertal SSC niche with functional Leydig and Sertoli cells (SCs) (de Michele et al., 2017a). However, the described culture media did not allow GC differentiation and a loss of spermatogonia (SG) was observed (de Michele et al., 2017b). The aim of this study was therefore to evaluate the possibility of achieving *in vitro* differentiation of human SSCs in organotypic culture of ITT, comparing two culture media enriched in molecules involved in GC self-renewal and differentiation. Indeed, compared to the culture media previously described (de Michele et al., 2017a), we used KSR as the only media that so far allowed a complete spermatogenesis with proven reproductive potential and we added GDNF (Glial cell derived neurotrophic factor) needed for SSCs self renewal (Chen and Liu, 2015), cholesterol to improve steroid production (Craig and Abdul, 2018) and prolactin and thyroid hormone to sustain SC maturation (Scarabelli et al., 2003), (Wagner et al., 2008). To our knowledge, this is the first report on IVM of cryopreserved human ITT showing *in vitro* GC differentiation up to and including the haploid stage.

## Materials and Methods

### Study Design

The study was designed to evaluate the possibility to achieve GC differentiation in frozen-thawed ITT retrieved from 5 patients and cultured in two different media for 139 days.

### Source of Human Tissue

Testis fragments were retrieved from 5 prepubertal patients aged 2–12 years participating to our fertility preservation program. The tissue used originated from deceased patients in order to allow culture of the tissue originally cryostored for clinical purposes. We excluded cases with history of urogenital diseases relevant to fertility and known genetic abnormalities. Prepubertal boys and their parents were referred before undergoing gonadotoxic treatments by pediatric oncologists who performed the pubertal clinical assessment (Tanner stages evaluation), which was further confirmed by the specialist in reproductive medicine at the moment of the discussion about the cryopreservation of the tissue. In all cases, an informed consent was signed by parents or legal guardians in order to cryobank testicular tissue both for clinical application and research purposes. Patients’ ages, pubertal state and diseases are reported in **Table 1**.

**Table 1:** Patients’ characteristics.

Patient	Age (years)	Pathology	Tanner stage	Most advanced germ cells in immature testicular tissue (before culture)	Johnsen score	Testicular volume
1	2	Medulloblastoma	P1G1	Spermatogonia	3	<4 cc
2	11	Medulloblastoma	P1G2	Spermatogonia	3	<4 cc
3	8	Neuroblastoma	P1G1	Spermatogonia	3	<4 cc
4	2	Pinealoblastoma	P1G1	Spermatogonia	3	<4 cc
5	12	Medulloblastoma	P1G1	Spermatogonia	3	<4 cc

*PI GI, Pubic hair:I, Genitalia: I; PIGII, Pubic hair:I, Genitalia: II.*

A fresh fragment fixed in paraformaldehyde (PFA 4%, VWR, Leuven, Belgium) at the moment of testicular biopsy and embedded in paraffin was available at the pathology department. The ethics review board of the Catholic University of Louvain approved all the experiments (Registration number B40320095364).

### ITT Processing

**FIGURE 1:**
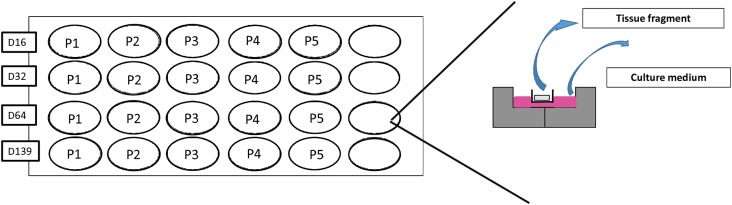
Culture plan. Tissue pieces of 1 mm^3^ were placed in wells of a 24-well plate inside Millicell inserts (Millipore – CM cell culture Inserts) half-soaked in 300 μl of culture media. A total of 20 tissue fragments were cultured per culture media, one per timing and per patient.

Frozen-thawed testicular tissue pieces were used for culture. Cryopreservation procedures using a DMSO based controlled slow-freezing protocol were already described (Wyns et al., 2007, 2008). After thawing, tissue was cut in pieces of 1 mm^3^, subsequently placed in wells of a 24-well plate inside Millicell inserts (Millipore—CM cell culture inserts, 12 mm/0.4 μm and cultured at 34°C in 5% CO_2_ with an air-liquid interface, according to the previously described protocol (Curaba et al., 2011; de Michele et al., 2017a) (for culture plan see **Figure 1**). The medium was changed every 48 h. Culture media, M1 and M2, are described in **Table 2**. Briefly, both culture media contained KSR and FSH 5 UI/L, while culture medium M1 was supplemented in GDNF, retinol, cholesterol, prolactin, T3, and hCG as factors responsible for SSCs self-renewal and differentiation.

**Table 2:** Culture media (M1 and M2) components.

Culture medium M1	Culture medium M2	Reference, country
KSR CTS^TM^ KnockOut^TM^ SR XenoFree Medium 10% in CTS^TM^ KnockOut^TM^ DMEM/F-12	KSR CTS^TM^ KnockOut^TM^ SR XenoFree Medium 10% in CTS^TM^ KnockOut^TM^ DMEM/F-12	ref. 12618013, Invitrogen, Belgium, Belgium (KSR) ref. A1370801, Invitrogen (DMEM)
FSH 5 UI/L	FSH 5 UI/L	Gonal F, Merck Serono, United Kingdom
Gentamicin 10 μg/mL	Gentamicin 10 μg/mL	ref. 15710-049, Invitrogen, Belgium
Ceftazidime 3 mg/L	Ceftazidime 3 mg/L	Kefadim, Biopharma, Italy
hCG 1 UI/L		Pregnyl, 1500 IU/mL, MSD, the Netherlands
L-Glutamin 0.35 mg/mL		Ref. 25030-024, Invitrogen, Belgium
Vitamin A (retinol) 10^-6^ M		Ref. R7632, Sigma-Aldrich, Belgium
Vitamin C 0.05 mg/mL		ref. A5960, Sigma-Aldrich, Belgium
Pyruvate 0.0025 M		Ref. P4562, Sigma-Aldrich, Belgium
5 pmol/L of triiodothyronine (T3)		Ref. 64245 Sigma Aldrich, Belgium
20 μM/L 22(R)-hydroxycholesterol		Ref. SC-205106A, Santa Cruz Biotechnology, United States
10 ng/mL GDNF		Ref. 212-GD-010, Bio-Techne, United States
5 ng/mL prolactin		Ref. L4021-50UG, Sigma Aldrich, Belgium

### Retrieval of Cultured Tissue Fragments and Supernatants

Cultured ITT fragments were harvested for analyses on Days 16, 32, 64, and 139 and then fixed in PFA 4% for 24 h before being embedded in paraffin. Five-micrometer-thick sections on Superfrost Plus slides (VWR, Belgium) were exploited for immunohistochemistry (IHC). Supernatants were retrieved every 2 days and stored at −20°C.

### Histological and Immunohistochemical Procedures and Analyses on Cultured Tissue

Immunohistochemistry and immunofluorescence (IF) were performed according to protocols previously described. All the slides for each marker were performed simultaneously in order to ensure standard conditions and homogeneous results. IHC analysis were performed on only one section/patient/condition (medium and timepoints)/marker (total of 40 sections per marker) due to scarcity of available tissue. Briefly, sections on Superfrost Plus slides underwent deparaffinization and rehydration. Endogenous peroxidase activity was blocked by incubation for 30 min with 0.3% H2O2. After washing in deionized water for 5 min, the slides were placed in citrate buffer for 75 min at 98°C. Thereafter, they were washed in 0.05 M Tris-buffered saline (TBS) and 0.05% Triton X-100 (Sigma-Aldrich) and, in order to block non-specific binding sites, they wereincubated at room temperature (RT) with 10% normal goat serum (NGS, Invitrogen) and 1% bovine serum albumin (BSA, Invitrogen) for 30 min (for 50 min for active caspase 3). The primary antibody (diluted to 1/500 for MAGEA4, 1/150 for Ki67, 1/200 for anti-Müllerian hormone (AMH), 1/150 for Glial cell line-derived neurotrophic factor (GDNF), 1/100 for the androgen receptor (AR), 1/400 for steroidogenic acute regulatory protein (STAR), 1/200 for active caspase 3, 1/4000 for BOLL, 1/4000 for SYCP3, 1/2000 for SOX9, 1/160 for Angiotensin-converting enzyme (ACE) and 1/2000 for Protamine 2, summarized in **Table 3**) was added to the sections and incubated overnight at 4°C in a humidified chamber (60 min for active caspase 3). Regarding double IHC for Ki67-MAGEA4, sections immunostained with anti-Ki67 were washed in HCl 0.1 M for 60 min, followed by washing in distilled water for 5 min and then in 0.05 M TBS and 0.05% Triton X-100 three times for 2 min each. Non-specific antibody binding was blocked by incubation of samples in 10% NGS and 1% BSA for 30 min at RT. MAGEA4 antibody was added to the samples and incubated at 4°C overnight in a humidified chamber.

**Table 3:** Antibodies and target cells.

Antibody	Target cell	Dilution	Manufacturer	Reference
ACE	Elongated spermatids	1/160	BIO-RAD	MCA2056
AMH	Mature Sertoli cells	1/200	Serotec	MCA2246
AR	Mature Sertoli cells	1/100	DAKO	AR441
BOLL	Spermatocytes and round	1/4000	Sigma-Aldrich	HPA 0488-13
CASPASE 3	Apoptotic cells	1/200	Promega	G7481
GDNF	Sertoli cells	1/150	Sigma-Aldrich	SAB1405863
Ki 67	Proliferating cells	1/150	DAKO	M7240
MAGEA4	Spermatogonia	1/500	Given by Giulio Spagnoli	/
PROTAMINE 2	Elongated spermatids	1/2000	Sigma-Aldrich	HPA056386
SOX9	Sertoli cells	1/2000	Abcam	185966
STAR	Leydig cells	1/400	Sigma-Aldrich	HPA027318
SYCP3	Spermatocytes	1/4000	Sigma-Aldrich	HPA039635
TNP1	Elongated spermatids	1/500	Sigma-Aldrich	HPA044387

The following day, the slides were washed in 0.05 M TBS and 0.05% Triton X-100 and the secondary anti-mouse antibody (EnVision+ System-Labeled Polymer-HRP; DAKO K4001) or anti-rabbit antibody (EnVision+ System-Labeled Polymer-HRP; DAKO, K4009) was added for an incubation time of 60 min at RT, followed by washing in 0.05 M TBS and 0.05% Triton X-100 three times for 2 min each.

The sections were then incubated for 5–10 min at RT with HRP-Green (Life Sciences, Germany) or the chromogen diaminobenzidine (DAKO K3468) and counterstaining of the nuclei with Mayer’s hematoxylinwas performed for 10 min. Eventually, the sections were dehydrated and mounted, to be scanned with a Leica SCN400 slide scanner (Leica Byosystem, WETZLAP, Germany).

For Immunofluorescence (IF), after blocking endogenous peroxidase activity with H2O2, the slides, after being washed in deionized water for 5 min, wereplaced in citrate buffer in microwave 900 watt for 4 min and 10 s, followed by 15 min at 90 watt and 1 min and 30 s at 900 watt. Then, the slides were washed in TBS/Tween (663684-B VWR Chemicals, Fontenay-sous-Bois, France) 0.1% for 5 min and non-specific antibody binding was blocked by incubation of samples with bovine serum albumin (BSA) 5% in TBS/tween. TNP1 antibody (dilution 1/500) was added to the slides and incubated 90 min at RT. The slides were washed in TBS/Tween and the secondary antirabbit antibody (DAKO K4003) was added for 40 min. After washing with TBS/tween 0.1%, fluorescein-coupled tyramide [(formed by tyramide hydricloride T2879-5G (Sigma, Lot BCBM4881V) and FITC-NHS: 21878-100 mg (Sigma, Lot BCBP2166V)] 1/200 diluted in Borate solution + 0.003% H2O2 was added to amplify the signal for 10 min at RT. Counterstaining was performed in Hoechst solution (Sigma, 12533–100 mg) 1/1000 for 5 min, and the slides were mounted after washing 10 min in TBS/Tween 0.1% with DAKO fluorescence mounting medium (DAKO, S302380). Images were acquired in structured illumination using a Zeiss AxioImager.z1 microscope, Göttingen, Germany, equipped with an ApoTome module (Carl Zeiss, Göttingen, Germany).

For positive IHC tissue controls, we used monkey ITT (for AMH), cerebellum (for GDNF), tonsil (for active caspase 3), mature human testicular tissue (for MAGEA4- Ki67, AR, STAR, BOLL, SYCP3, TNP 1, ACE, and Protamine 2). For negative controls the primary antibody was omitted.

Only well-preserved STs (integrity score 3 and4) were taken into account for IHC analyses.

### Tissue Histological and Immunohistochemical Evaluation

#### Seminiferous Tubule Integrity

We performed a double blind evaluation (MV and FdM., slides analyzed without knowledge of the timepoint and the patient). A semi-quantitative analysis of the integrity of the STs under light microscopy at ×400 magnification was performed using a score previously described (de Michele et al., 2017a). We counted for the histological analyses 4 slides for each timepoint and culture media, picked per every fourth slide. Four parameters were assessed: adhesion of cells to the basement membrane, cell cohesion, proportion of pyknotic nuclei (less than 5% of total), and easy distinction of germ cells and Sertoli cells. The score ranged from 1 to 4 (tubules with the worst to best integrity, respectively).

#### Spermatogonial Survival and Intra-Tubular Cell Proliferation

Anti-human mouse MAGEA4 monoclonal antibody (kindly given by Giulio Spagnoli, MD) was used to identify SG (Yakirevich et al., 2003). Results were presented as the mean quantity of MAGEA4-positive cells per ST. Anti-human mouse Ki67 monoclonal antibody (DAKO M7240, Heverlee, Belgium) was exploited to evaluate intratubular proliferation (Scholzen and Gerdes, 2000) and the mean amount of Ki67-positive cells per ST was recorded.

Double-stained MAGEA4/Ki67-positive cells identified proliferative SG and proliferative SCs were counted as the difference between the total amount of intratubular Ki67-positive cells and proliferative SG. The ratio of proliferative SG was calculated as the ratio between the number of proliferative SG and the total number of SG.

#### Cell Apoptosis

Active caspase 3 rabbit anti-human polyclonal antibody (Promega G7481, the Netherlands) was used to study apoptotic cells (Elmore, 2007). The ratio between active caspase 3-stained cells and the total number of well-preserved tubules was calculated.

#### SC Viability, Functionality, and Maturation

The number of SCs per ST was counted using SOX9 staining (Abcam 185966) (Frojdman et al., 2000). Anti-human mouse GDNF polyclonal antibody (Sigma-Aldrich SAB1405863) identified viable SCs able to produce GDNF needed for spermatogonial self-renewal (Davidoff et al., 2001), and its staining was evaluated as “presence/absence” in STs. AR mouse anti-human monoclonal antibody (DAKO, AR441) for the AR was used as a marker of SC maturation (Rey et al., 2009). AMH mouse anti-human monoclonal antibody (Serotec MCA2246) evidenced the presence of AMH (using a score given to each ST based on its intensity of expression from “strong” to “absent”), a marker of SC maturation since its expression is strong in immature SCs and diminishes at the onset of puberty (Sharpe et al., 2003). SC maturation was further assessed via AMH secretion in supernatants of culture media retrieved at every culture medium replacement (every 48 h) using an ultra-sensitive enzyme-linked immunosorbent assay (ELISA; Ansh Labs, Webster, TX, United States, limit of detection 23 pg/mL, dynamic range 6, 0.084–14.2 ng/mL) (After dilution 5×) (Gruson and Homsak, 2015).

#### Germ Cell Differentiation

The histological morphological aspect of differentiating GCs was assessed following the description by Clermont (1972). According to this description, spermatogonia are adjacent to the basement membrane and easily identifiable by a typical white cytoplasmic halo around the nucleus. Primary spermatocytes are characterized by a large nucleus and very thick and short chromatin strings. Newly formed spermatids are localized in the adluminal compartment of STs, are small in size, pear- or paddle-shaped, with a small spherical nucleus containing condensed chromatin. IHC for SYCP3 (identifying spermatocytes) and BOLL (identifying spermatocytes and round spermatids) were performed (Yuan et al., 2000; Kostova et al., 2007). IHC for protamine 2 and ACE, and IF for TNP1 were used to evidence elongated spermatids (Meistrich et al., 2003; Pauls et al., 2003; Eirin-Lopez et al., 2006).

### Haploid Cell Evaluation Using Chromogenic *in situ* Hybridization (CISH)

Haploid cells were identified by CISH as two spots, one in red and one in black, identifying chromosome 17 and human epidermal growth factor receptor 2 (HER2), respectively, counted and expressed as the number of cells per ST.

The CISH procedure is routinely applied for the establishment of HER2 ploidy in biopsies from cancer patients in our institution using the platform BenchMark GX, Ventana, Roche. The slides are hybridized with the specific probes for HER2 and chromosome 17 (INFORM Her2 DUAL ISH DNA Cocktail Probe, ref. 800-4422, Ventana, Roche), which contains the HER2 probe labeled with dinitrophenyl (DNP) and the chromosome 17 probe labeled with digoxigenin (DIG) formulated with human placental blocking DNA in a formamide based hybridization buffer.

Detection of the DNP-labeled HER2 probe occurs first using the detection systems UltraView Red ISH Dig Detection Kit (ref.:800-505, Ventana, Roche). Following CISH detection for HER2, the DIG-labeled chromosome 17 probe is detected via the UltraView SISH DNP Detection Kit (ref.:800-098, Ventana, Roche). The specimens are then counterstained with Hematoxylin.

The tissue sections are hybridized with specific probes for HER2 and chromosome 17 (INFORM Her2 DUAL ISH DNA Cocktail Probe, ref. 800-4422, Ventana, Roche). The slides were scanned with a Leica SCN400 slide scanner (Leica Byosystem, WETZLAP, Germany) in 7 layers of 0.5 μm of thickness in order to avoid false positive errors in assessment of haploid cells.

### Leydig Cell Functionality

LC functionality was assessed by testosterone measurement in supernatants using electro-chemiluminescence immunoassays (ECLIA; Roche, Brussels, Belgium) after dilution 100× and by IHC for STAR (Luo et al., 2001) counting the number of STAR-positive cells in three fields analyzed consecutively from the left corner to the right at ×400 magnification.

### Statistical Analysis

The impact of the medium and time were studied using a mixed-effect linear regression model. In this model, the patient effect was supposed to follow normal distribution and was then defined as a random effect, while time and medium were taken into account as fixed effects. An interaction effect between time and medium was included in each model to investigate whether the medium effect was similar between each time point, and when non-significant (*p* > 0.05), this interaction effect was removed from the model. The global effect of time was evaluated on each outcome and the different timepoints were thus not compared. Moreover, for each outcome, we evaluated if a log-transformation was required in order to meet the assumptions of the statistical model (i.e., residuals with normal distribution and homogeneity of variance). We used the software R, Version 3.4.0 R Foundation for Statistical Computing, Vienna, Austria.

Results were expressed as mean ± SD for all five patients. The results were considered as statistically significant when the *P*-value was lower than 0.05.

## Results

### Tubular Integrity

**FIGURE 2:**
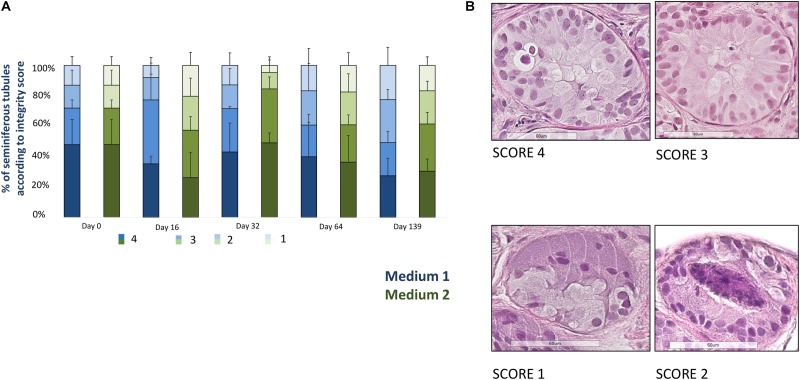
Tissue integrity. **(A)** Shows the evolution of seminiferous tubules (STs) integrity for each culture time point, represented as the percentage of STs classified according to a scoring system where score 4 corresponds to the best STs integrity and score 1 to the worst integrity. Four parameters were used to assign a ST to a given score: adhesion of cells to the basement membrane, cell cohesion, proportion of pyknotic nuclei (less than 5% of total), and easy distinction of germ cells and Sertoli cells. The histogram bars colored in blue correspond to medium 1 and those in green to medium 2. A statistical significant reduction (*p* = 0.02) of the well preserved STs (score 3 + 4), evaluated in four slides for each timepoint and each culture media in all the patients, was observed, without any difference between the two culture media. **(B)** Illustrates four examples of tubules from the best to the worst score (from 4 to 1). The tubule reported as score 4 shows an easy distinction between Sertoli cells and germ cells, a good cohesion and adhesion of cells to the basement membrane, while the tubule with a score 3 contains pyknotic nuclei within the lumen. The tubule reported as a score 2 presents pyknotic nuclei within the lumen and lack of cell cohesion, while the score 1-tubule shows necrosis at the middle of the tubule and lack of cell cohesion and adhesion.

**Figure 2** shows the evolution of the proportion of well-preserved STs (score 3 + 4). A total of 2831 well-preserved STs was analyzed (1713 for medium 1 and 1118 for medium 2, mean of 20.5 ± 14.6 STs per condition). At day 139 the percentage of well-preserved STs was 56.9% for M1 and 85.5% for M2, with a statistical significant reduction along the culture (*p* = 0.02) and without any statistical difference between the two media.

### Spermatogonial Survival and Intra-Tubular Cell Proliferation

**FIGURE 3:**
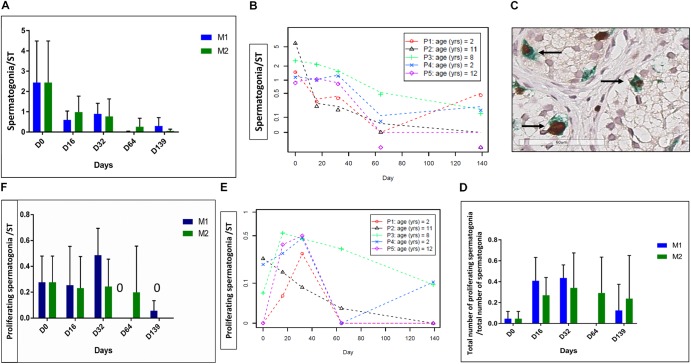
Spermatogonia survival and proliferation. The figure shows the survival and proliferation of spermatogonia (SG) at the different culture timepoints. **(A,F)** Shows the results -expressed as the mean ± SD between the five patients- of the spermatogonial number per ST **(A)** and of the number of proliferative SG, identified as double-stained MAGE-A4 and Ki67-positive cells per tubule **(F)**. Moreover, as no statistical difference was found between the two culture media, the same data are reported also for each patient, as a mean between the two media in **(B,E)**. A statistically significant reduction was observed for both parameters (*p* ≤ 0.001 for the spermatogonial number per ST and *p* ≤ 0.01 for the number of proliferative SG per ST). The ratio of proliferative SG, calculated as the ratio between the number of proliferative SG and total number of SG, is shown in **(D)**, and it did not change during the culture period. **(C)** Shows the double staining MAGE A4-Ki67 where proliferative spermatogonia are pointed by an arrow.

A total of 1046 STs were analyzed (578 for M1 and 468 for M2, mean of 24.9 ± 15.1 STs per condition). Spermatogonia were maintained over the whole culture period. The evolution of MAGEA4 positive cells is shown in **Figures 3A,B**, and a statistically significant reduction of the mean number of SG per ST (*p* ≤ 0.001) was observed. The ratio of proliferative SG did not change (**Figure 3D**), while the number of proliferating SG per ST decreased over the culture period (*p* ≤ 0.01) (**Figures 3E,F**).

**FIGURE 4:**
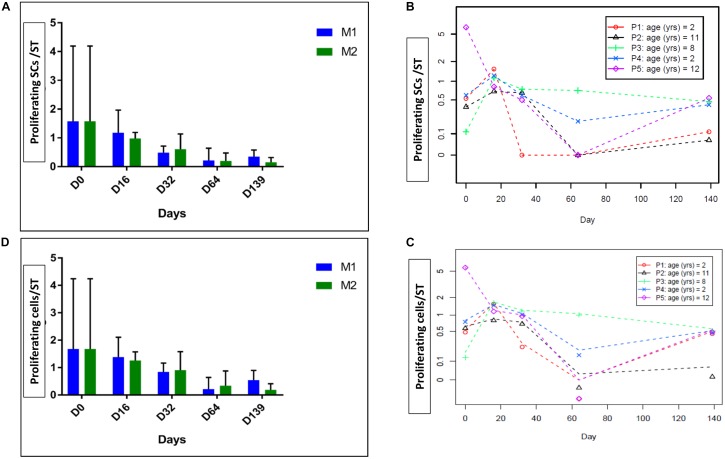
Intratubular cell proliferation. The figure shows the proliferation of intratubular cells and proliferating SCs at the different culture timepoints. **(A)** Shows the evolution of proliferating SCs per ST and **(D)** shows the evolution of proliferating cells per ST, both parameters expressed as a mean between the five patients. **(B,C)** Shows the same results expressed for each patient, where data are presented as a mean between the two culture media as no statistical difference was found between the two media. We observed a statistically significant reduction in the number of proliferative SCs over the culture period (*p* = 0.04) and in total intratubular Ki67-stained cells (*p* = 0.04).

Spermatogonia and proliferative cells are illustrated in **Figure 3C**. The number of proliferative SCs per ST decreased over the culture period (*p* = 0.04) (**Figures 4A,B**), as did the total Ki67-stained cells per ST (*p* = 0.04) (**Figures 4C,D**). No statistical difference was found between the two culture media.

### Cell Apoptosis

**FIGURE 5:**
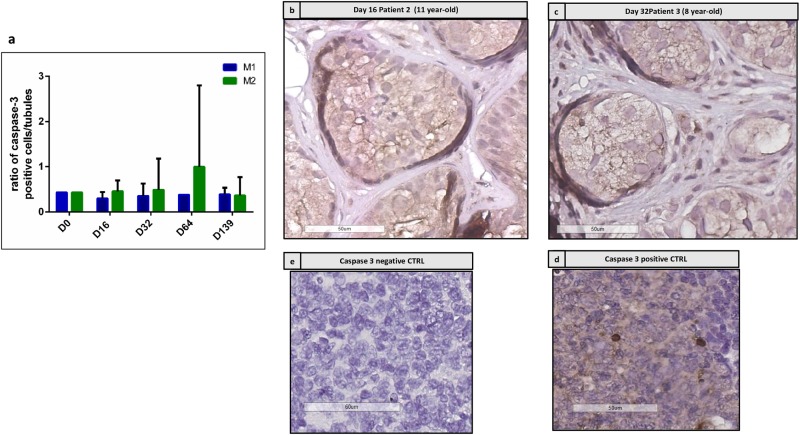
Active caspase 3 immunostaining. **(a)** Shows the evolution of intratubular cells stained for active caspase 3, which did not show any significant change along the culture. **(b,c)** Show the staining of caspase 3 in patient 2 at day 16 **(b)** and for patient 3 at day 32 **(c)**, where no stained cells were detected in the adluminal compartment of corresponding STs presented on HE sections in **Figures 8c,d**. Some positive cells are found at the basement membrane. **(d,e)** Show negative and positive controls for caspase 3 performed on tonsil tissue.

**Figure 5** shows the immunostaining for active caspase 3. **Figure 5a** shows the ratio between active caspase 3-positive cells and the total number of well-preserved tubules, which did not change along the culture. No difference was found between the two culture media. **Figures 5b,c** show the IHC performed on the same tubules already reported in **Figures 8c,d**, where no caspase 3 positive cells were stained in the adluminal compartment, while positive cells are present at the basement membrane.

### SC Viability and Maturation

**FIGURE 6:**
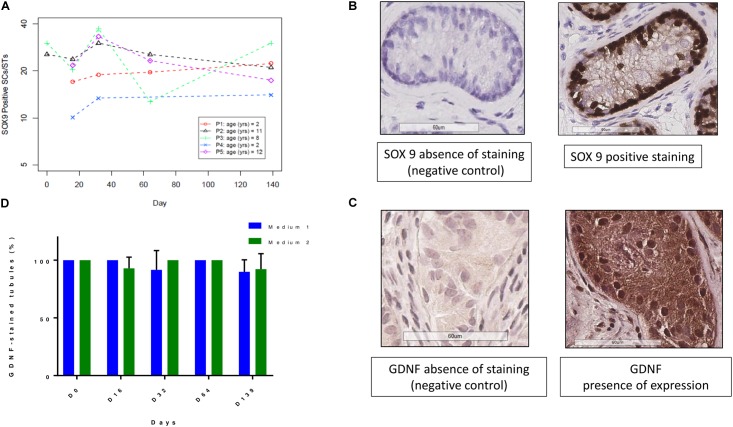
GDNF and SOX9 expressions at the different culture timepoints. **(A)** Shows the evolution of SOX9 positive SCs, expressed for each patient as a mean between the two culture media. The number of SOX9 positive SCs did not change along the culture period. **(B)** Reports the images of SOX9 staining and negative control, where the primary antibody was omitted. **(D)** Demonstrates that GDNF (glial cell line-derived neurotrophic factor) expression, calculated as the ratio between stained STs and the total number of STs, did not change over the course of culture for both culture media. The culture media are represented in blue for M1 and in green for M2. **(C)** Show images of presence and absence of GDNF IHC expression.

**FIGURE 7:**
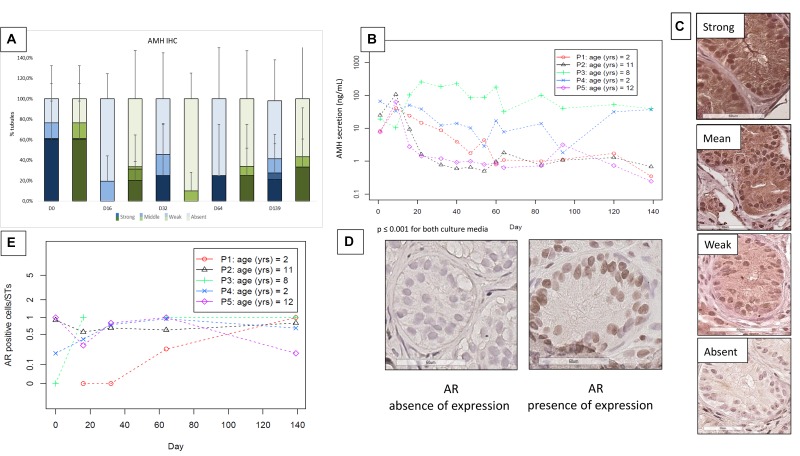
Anti-Müllerian hormone (AMH) expression and secretion (**Figures 5a–c**) and androgen receptor (AR) expression (**Figures 5d,e**). **(A)** Illustrates IHC expression of AMH, evaluated via a semiquantitative score, where the darkest colors represent the strongest staining of the marker, with a progressive decrease in color intensity corresponding to the decrease in IHC staining (in blue for medium 1 and in green for medium 2). The “strong and moderate” expression of AMH showed a trend in diminution, without significant change. **(B)** Shows AMH secretion in the supernatants, where – as no statistical difference was found between the two culture media- the data are reported for each patient as a mean between the two media, with a significant decrease during the culture period (*p* ≤ 0.001). **(C)** Shows the different intensity of staining of AMH from strong (in the top) to absent (at the bottom of the figure). The images were taken from the strong to the absent staining in patient P2 day 16, P1 day 64, P5 day 64, and day 139, respectively. **(E)** Illustrates the evolution of AR stained cells per ST, which did not show a significant change along the culture. We reported the values for each patient, calculating the mean between the two culture media as they did not show any statistical difference. The absence and presence of AR expression is illustrated in **(D)**.

The evolution of SOX9-positive cells during culture is shown in **Figures 6A,B**. A total of 753 STs were analyzed (378 for M1 and 375 for M2, mean of 23.5 ± 16.1 STs per condition). There was no statistically significant variation in the number of SOX9-positive cells per ST. A total of 1094 STs both for GDNF and AMH was analyzed (595 STs for M1 and 499 for M2), with a mean of 26.7 ± 17.1 STs per condition. **Figures 6C,D** show the GDNF staining up to day 139 with a range of variation of stained STs from 100 to 90% in both culture media. AMH expression (**Figure 7C**) showed a trend in diminution although it did not reach statistical significance (**Figure 7A**). AMH secretion in the supernatants showed a significant progressive decrease during the culture period (*p* ≤ 0.001) (**Figure 7B**).

**Figures 7E,D** show AR IHC (**Figure 7D**) and its evolution during the culture period for each patient (**Figure 7E**), which did not show any variation during the culture. A total of 827 STs was analyzed (401 for M1 and 426 for M2, mean of 22.3 ± 14.6 STs per condition).

### Germ Cell Differentiation

#### Histological Analyses

**FIGURE 8:**
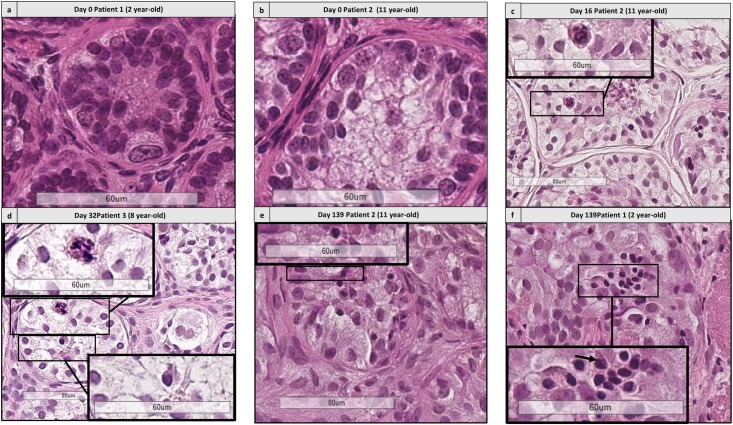
Histological assessment of differentiating germ cells. **(a,b)** Shows the presence of spermatogonia from patient 1 aged 2 years old and patient 2 aged 11 years old. **(c–f)** Shows the presence of differentiating germ cells at early culture time points (D16 and 32) and late time points (D139) for patient 2 **(c,e)** aged 11 yearss old, for patient 3 aged 8 years old **(d)** and patient 1 aged 2 years old, culture medium 2 **(f)**. Cutouts show the differentiating germ cells at a higher magnification, like a spermatocyte with a characteristic granulo-filamentous chromatin **(c,d)**, and round spermatids with the small condensed and eccentric nucleus, adluminal localization and pear shape **(d–f)**.

**FIGURE 9:**
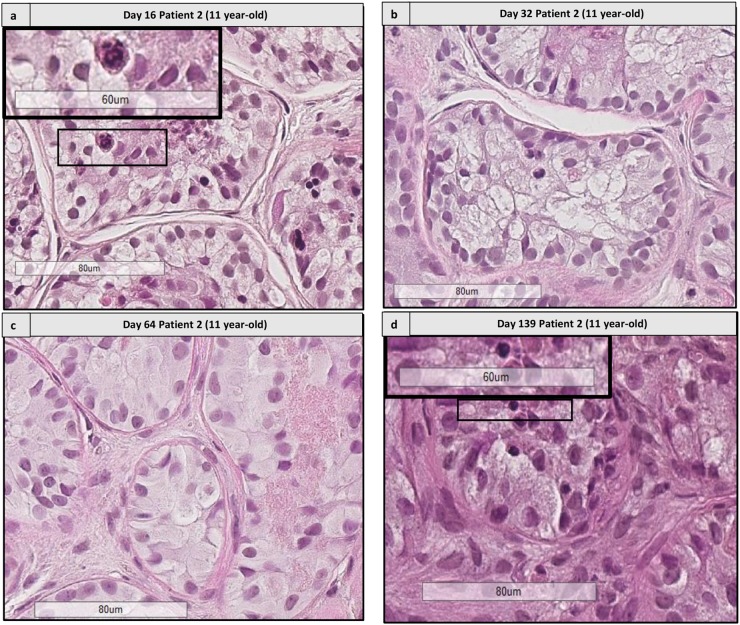
Histological assessment of sequential images from the same patient. This figure shows the histological images from patient 2 (aged 11 years old) from days 16 to 139. **(a,d)** Shows the presence of differentiating cells at days 16 and 139, respectively. Cutouts show the differentiating germ cells at a higher magnification, like a spermatocyte with a characteristic granulo-filamentous chromatin **(a)**, and round spermatids with the small condensed and eccentric nucleus, adluminal localization and pear shape **(d)**. In the timepoints 32 and 64 **(b,c)** we did not find any differentiating cells, as different fragments were harvested for the different timepoints.

Differentiating GCs corresponding to spermatocytes and spermatids, according to the description by Clermont (Clermont, 1972; **Figures 8**, **9**) were observed from day 16, for both culture media. A total of 2831 STs was analyzed (1713 for M1 and 1118 for M2, mean of 20.5 ± 14.6 STs per condition).

#### IHC and IF Staining

**FIGURE 10:**
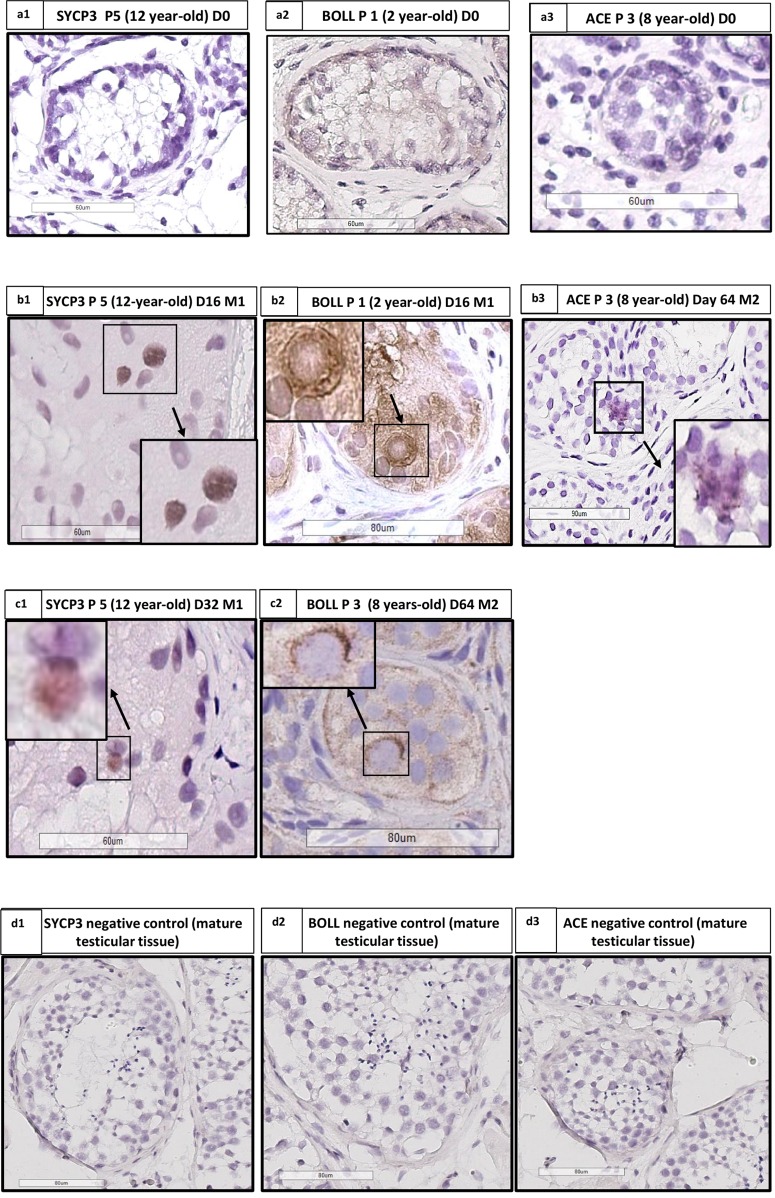
Angiotensin converting enzyme (ACE), Synaptonemal complex protein 3 (SYCP3) and Protein boule-like (BOLL) expression. **(a1,b1,c1)** Show IHC for SYCP3 in patient 5 (aged 12 years old), at days 0, 16, and 32, respectively, with the presence of staining at day 16 **(b1)** and day 32 **(c1)**. **(a2,b2,c2)** Show IHC for BOLL in patient 1 aged 2 years old, at days 0, 16, and 64, respectively, with the presence of staining at days 16 and 64. **(a3,b3)** Shows IHC for ACE in patient 3 aged 8 years old at day 0 (absence of staining) and 64 (presence of staining). **(d1–d3)** Shows the SYCP3, BOLL, and ACE negative controls performed on mature testicular tissue. We analyzed one slide for each patient and for each time point, for both culture media.

Few cells stained for SYCP3 (spermatocytes), BOLL (spermatocytes and round spermatids) and ACE (elongated spermatids) were observed in cultured samples of patients at different ages from day 16 onwards as shown in **Figure 10**. BOLL-stained cells were found in all the sections and all conditions (40/40 sections), while SYCP3, as a more transient protein, was detected in 2/40 sections (in patient 5 at day 16 and 32). ACE was found only in patients 8 at day 64 (1/40 sections). We did not observe any staining for TNP1 and protamine 2. We analyzed a total of 805 STs (430 for M1 and 375 for M2, mean of 21.7 ± 15.5 STs per condition).

#### Haploid Cell Evaluation via CISH

**FIGURE 11:**
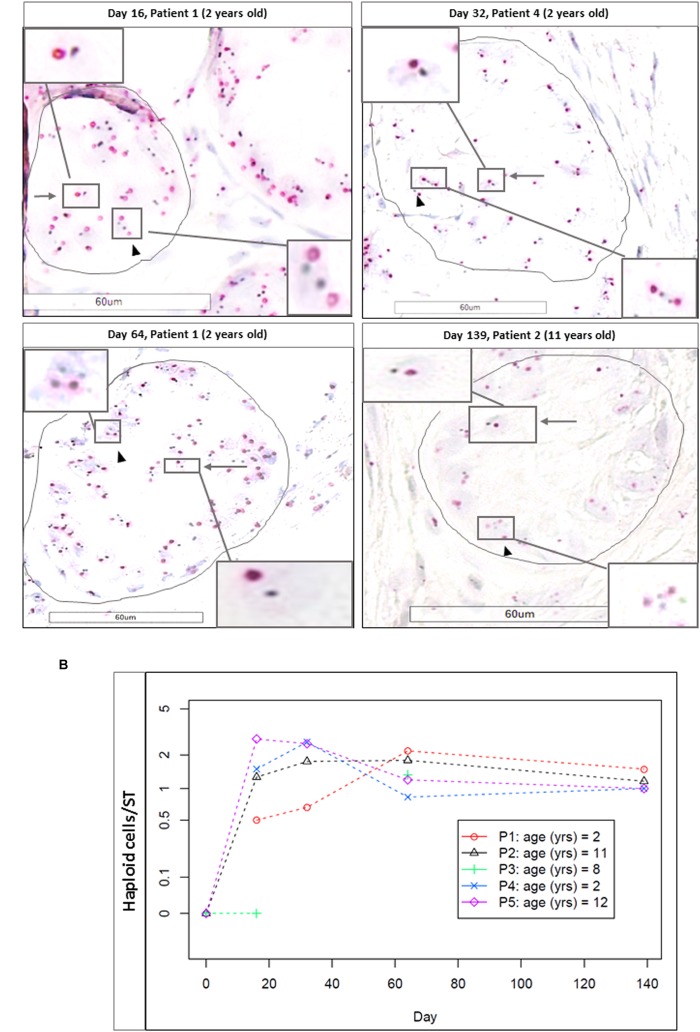
Haploid cells evidenced zzby CISH. **(A)** Shows the CISH-stained cells (magnification ×400) at different timepoints (D16, D32, D64, and D139). Arrows indicate haploid cells, identified as two stained spots (in red for Ch17 and in black for HER2). Arrow heads show diploid cells, characterized by the presence of four spots. Cutouts give images at higher magnification (×600). Haploid cells were found in the adluminal compartment, while diploid cells were found on the basement membrane, underlined in black. **(B)** Shows the number of the haploid cells per ST for each patient, and as no differences were observed between the two culture media, we reported the results as a mean between M1 and M2. No haploid cells were found at day 0.

Haploid cells were evaluated in a total of 347 STs (184 for M1 and 163 for M2, mean of 10.5 ± 8.2 STs per condition). Each time point (D16, D32, D64, and Day 139) showed a significantly higher number of haploid cells per ST, compared to the initial time point (D0, taken as reference, where no haploid cells were observed; *p* ≤ 0.003 for D16, *p* ≤ 0.008 for D32 and D64, and *p* ≤ 0.001 for D139). **Figure 11A** shows CISH-stained cells and **Figure 11B** the number of haploid cells per ST. We did not find any statistical difference in the number of haploid cells per ST between days 16 and 139 for either of the two culture media.

#### Correlation Between Haploid Cells and Spermatogonial Cells

We found a statistical correlation between the increase in the number of haploid cells per ST using CISH and the decrease in MAGE A4-positive cells per ST (*p* = 0.02).

### Leydig Cell Functionality

**FIGURE 12:**
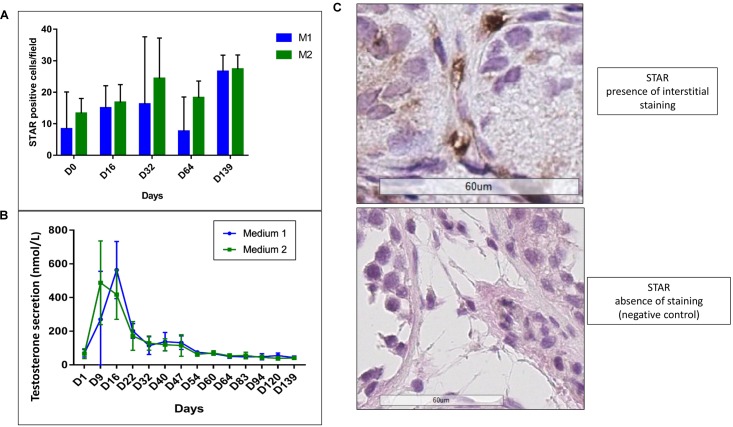
Leydig cell functionality. **(A)** Shows the increase (*p* ≤ 0.007) in STAR IHC expression, without any difference between the two culture media (M1 in blue and M2 in green). The interstitial staining (showed in **B**) was evaluated counting the number of STAR-positive cells in three consecutive-fields at × 400 magnification from the left corner to the right, in order to standardize and homogenize the counting system. The graph **(C)** shows a peak in testosterone production at day 15 up to 563 nmol/L (M1, in blue) and 417 nmol/L (M2, in green), with a statistical significant decrease (*p* ≤ 0.001) of the secretion along the culture and a stabilization at day 64. No difference between the two culture media was observed.

We observed a statistical increase in STAR expression (*p* ≤ 0.007) (**Figures 12A,B**). A peak in testosterone secretion up to 563 nmol/L (M1) and 417 nmol/L (M2) at day 16 and a significant reduction along the culture (*p* ≤ 0.001) with stabilization from day 64 at 43 nmol/L (M1) and 40 nmol/L (M2) were observed (**Figure 12C**). No difference between the two culture media was observed for both analyses.

## Discussion

We have described an organotypic culture system for human ITT developed to obtain *in vitro* differentiation of GC, applying a system that previously only allowed preservation of ST integrity, SG survival, SC maturation and LC function (de Michele et al., 2017a). Using different media, we obtained GC differentiation up to round spermatids after 16 days of culture.

Spermatogenesis is a stepwise series of events starting from SSCs that self-renew and are committed to differentiation: primary spermatocytes undergo meiosis and differentiate into secondary spermatocytes and round spermatids, followed by spermiogenesis that leads to the formation of spermatozoa. The entire process lasts 64–74 days in humans (Djureinovic et al., 2014). The presence of postmeiotic cells in our culture system after few days of culture points to a receptiveness of ITT which, in specific culture conditions and media, is able to start quickly SC maturation and GC differentiation.

We compared two culture media, with the objective to confer ideal conditions to the culture system that reproduce the niche microenvironment physiologically present in the testis at the onset of puberty and during the pubertal transition period. Besides FSH addition in both media at a concentration of 5 UI/L, in one medium (M1) some molecules known to play a role in GC survival and differentiation were added, like hCG, which stimulates the production of testosterone, known as a differentiation and survival factor for GC (Sharpe, 1987). Moreover, M1 was also supplemented with retinoic acid (RA), which has been exploited in culture of human adult tissue with achievement of meiosis (Yang et al., 2014; Perrard et al., 2016; Sun et al., 2018). However, while addition of RA to the culture media did not allow GC differentiation in our previous work despite SCs maturation (de Michele et al., 2017a), GC differentiation was obtained in this work in M2 in the absence of RA. These observations raise questions on the role of KSR used as basic medium here and on the amount of FSH added (5 IU/L here versus 50 IU/L previously (de Michele et al., 2017a). Indeed, while supplementation of culture media with 5 UI/L FSH, which is a physiological serum concentration, seems to be able to induce meiosis, as shown by the presence of differentiating germ cells after 16 days of culture in both culture media, higher concentration did not allow GC differentiation, as found in our previous work (de Michele et al., 2017a). A possible explanation of the inability of FSH at a concentration of 50 UI/L to induce meiosis could be the desensitization of FSH receptors and of adenyl cyclase (with consequent reduced cAMP production) in SCs (Jahnsen et al., 1982), which could make them unable to sustain spermatogenesis *in vitro*. A further corroboration of the plausibility of this hypothesis is provided by the study of Tesarik on adult testicular tissue (Tesarik et al., 2000a), where concentrations of FSH higher than 20 UI/L added to the culture media were related with poor prognosis in terms of *in vitro* differentiation potential of GCs.

The ignorance of the actual content of KSR, known to induce complete spermatogenesis in immature mice testicular tissue (Sato et al., 2011; Yokonishi et al., 2014; Dumont et al., 2015) precludes drawing further conclusions on media content and points to the necessity to disclose culture media components. In addition, regardless of medium composition, organotypic culture systems may also play a role as Sato et al. (2011) used a different system from ours where germ cells retrieved from a donor mice were cultured for 6–9 weeks and transplanted into a host mice, whose testis tissue was then harvested and cut in pieces, before being organotypically cultured in agarose gel stands half-soaked in the culture medium. Moreover, the same team also further developed this organotypic culture system using a microfluidic device able to sustain spermatogenesis and generate healthy mice offspring, opening the way to a new concept of dynamic organotypic culture, where nutrients and oxygen are able to flow separately from the tissue diffusing through a porous membrane (Komeya et al., 2016; Yamanaka et al., 2018).

Although we found meiotic and postmeiotic cells after 16 days of culture as indicated by the presence of SYCP3 and BOLL positive cells as well as haploid cells using CISH, we did not observe spermiogenesis as shown by the absence of more advanced differentiation markers (TNP1 and protamine 2). The presence of ACE positive cells in a patient aged 8 years old suggests that there might be a different capability of ITT of different ages to undergo differentiation, but the scarcity of tissue available for research did not allow to confirm this hypothesis.

The presence of fewer cells at IHC compared to CISH could be due either to the transient phases of morphological aspects of cells, which maintain their haploid status (stated by the CISH) while progressing through differentiation, or to a delay in the expression of differentiation proteins compared to the genetic modifications. The genetic status of the cells does not undergo variation along time, while the intensity of the expression of the proteins, evaluated by IHC, can vary during the culture time. Moreover, IHC and CISH were not performed on subsequent slides, which can imply that the targeted cells are different.

A further limitation of this study is that we cannot completely rule out that some differentiated cells could be present in pre-cultured tissue, as it is well known that in prepubertal testis spermatogenesis waves can be physiologically present ending subsequently in germ cell apoptosis. However, no markers of haploid cells were found in pre-cultured fragments and the statistical significant increase in haploid cells also supports the evidence of development of haploid cells during culture. Moreover, the absence of active caspase 3 stained cells in the adluminal compartment of tubules where meiotic GCs were found from 16 days of culture onwards is a further confirmation of the development of newly differentiated cells during culture.

The lack of spermatid elongation raises the question about the ability of the testicular niche to preserve its functionality *ex vivo* and to sustain complete spermatogenesis. In addition, *in vitro* cells requirements, i.e., effective concentrations of media components and their mutual interactions should be further elucidated. Indeed, as FSH plays a fundamental role in elongation and spermiation, the lack of elongation of differentiating germ cells could be due to an insufficient concentration of FSH required for spermiogenesis (Matthiesson et al., 2006; Ruwanpura et al., 2010). Furthermore, FSH also regulates the LCs production of testosterone (Sriraman and Jagannadha Rao, 2004) known to play an important role in spermiogenesis (Matthiesson et al., 2006; Sofikitis et al., 2008). Although the FSH concentration in our culture media (5 UI/L), chosen according to its physiological blood concentration in men (Ruwanpura et al., 2010), was able to induce *in vitro* meiosis, such concentration could have been insufficient to ensure an efficient production of testosterone *in vitro*. Even if LCs functionality was shown during the whole culture period by the expression of STAR and continuous production of testosterone, we observed a significant decrease of its secretion from 563 nmol/L (M1) and 487 nmol/L (M2) to 43 nmol/L (M1) and 40 nmol/L (M2), below the physiological intratesticular concentration of about 600 nmol/L (Ruwanpura et al., 2010). This could potentially account for the observed blockade at the round spermatid stage (Matthiesson et al., 2006; Sofikitis et al., 2008). The lack of a statistical difference in testosterone secretion between the two media, where only M1 was enriched with hCG for its LH-like activity to sustain LCs function, is intriguing and further supports the role of FSH in the induction of testosterone production (Sriraman and Jagannadha Rao, 2004). Moreover, when a higher amount of FSH (de Michele et al., 2017a) was added in the same culture system (where KSR was absent and either hCG or exogenous testosterone were added) a 10-fold higher concentration of testosterone was reached. We may, however, not exclude that the reduction in testosterone could be ascribed to some kind of exhaustion of the LCs during the culture, although this is less likely considering the increased expression of STAR and also previously reached testosterone levels at day 139 in the same culture system (de Michele et al., 2017a).

The maturation of SCs was shown by a statistically significant reduced secretion of AMH, further corroborated by the SC proliferation arrest reached in mature SCs (Sharpe et al., 2003). AR expression only showed a trend to increase (not statistically significant) but variation of AR expression linked to differences in tissue receptivity related to patients’ ages may limit the interpretation of this result.

Although the number of haploid cells reached per ST remained constant during the culture, it is not excluded that the culture system fails in achieving full maturation of somatic cells needed for SSC renewal and for accomplishing complete differentiation. Culture conditions may also be responsible for impaired tissue homeostasis with unbalance between renewal and differentiation as suggested by the correlation between the increase in haploid cell numbers and the reduction in SG. Indeed, on the one hand, the presence of haploid cells at 16 days seems an accelerated process compared to the postpubertal physiological maturation in humans which takes about 24 days to proceed from the preleptotene stage to the formation of round spermatids (O’Donnell et al., 2000). On the other hand the observed depletion of the spermatogonial pool could be attributed to failure in the renewal process despite supplementation of culture media (M1) with GDNF, a SC secreted self-renewal factor for spermatogonia acting on the GFRα receptor. Exhaustion of SCs due to culture conditions cannot be ruled out although the constant presence of AR and the maintenance of SOX9 positive cells does not call for this hypothesis. Further studies in order to clarify the GDNF pathway and to achieve a better knowledge of its action are required.

## Conclusion

We demonstrated for the first time the differentiation of GCs up to a postmeiotic stage when slow-frozen-thawed prepubertal testicular tissue from patients of different ages were cultured, representing a relevant milestone in the field of fertility preservation for prepubertal boys. The maturation process seemed to be accelerated when related to the progressive and gradual *in vivo* niche maturation (Marshall and Tanner, 1970), suggesting that *in vitro* culture could shorten the *in vivo* time period of peripubertal modifications within the testicular microenvironment. In addition, GC differentiation seems to be accelerated compared to the postpubertal physiological maturation (O’Donnell et al., 2000). Besides work to achieve spermatid elongation, further evidence of the genetic and epigenetic normality of the haploid cells must still be demonstrated but the scarcity of available tissue to perform long-term culture with interim assessments and the low efficiency of the meiotic process precluded additional analyses.

## Author Contributions

FdM ideated the project, performed the experiments, analyzed the results, and wrote the manuscript. JP participated to the ideation the project and contributed to the analysis of the results. MV helped performing experiments and participated to the discussion. JA conducted the statistical analyses. DG performed the supernatant analyses. YG performed the CISH analyses. CW was responsible for the project concept, interpretation of results, critical discussion, and review of the manuscript.

## Conflict of Interest Statement

The authors declare that the research was conducted in the absence of any commercial or financial relationships that could be construed as a potential conflict of interest.
